# Efficient big data assimilation through sparse representation: A 3D benchmark case study in petroleum engineering

**DOI:** 10.1371/journal.pone.0198586

**Published:** 2018-07-27

**Authors:** Xiaodong Luo, Tuhin Bhakta, Morten Jakobsen, Geir Nævdal

**Affiliations:** 1 International Research Institute of Stavanger (IRIS), Bergen, Norway; 2 University of Bergen, Bergen, Norway; PLOS, UNITED KINGDOM

## Abstract

Data assimilation is an important discipline in geosciences that aims to combine the information contents from both prior geophysical models and observational data (observations) to obtain improved model estimates. Ensemble-based methods are among the state-of-the-art assimilation algorithms in the data assimilation community. When applying ensemble-based methods to assimilate big geophysical data, substantial computational resources are needed in order to compute and/or store certain quantities (e.g., the Kalman-gain-type matrix), given both big model and data sizes. In addition, uncertainty quantification of observational data, e.g., in terms of estimating the observation error covariance matrix, also becomes computationally challenging, if not infeasible. To tackle the aforementioned challenges in the presence of big data, in a previous study, the authors proposed a wavelet-based sparse representation procedure for 2D seismic data assimilation problems (also known as history matching problems in petroleum engineering). In the current study, we extend the sparse representation procedure to 3D problems, as this is an important step towards real field case studies. To demonstrate the efficiency of the extended sparse representation procedure, we apply an ensemble-based seismic history matching framework with the extended sparse representation procedure to a 3D benchmark case, the Brugge field. In this benchmark case study, the total number of seismic data is in the order of $O(10^{6})$. We show that the wavelet-based sparse representation procedure is extremely efficient in reducing the size of seismic data, while preserving the salient features of seismic data. Moreover, even with a substantial data-size reduction through sparse representation, the ensemble-based seismic history matching framework can still achieve good estimation accuracy.

## Introduction

Data assimilation is an important discipline in geosciences that aims to combine the information contents from both prior geophysical models and observational data (observations) to obtain improved model estimates [1]. The advance of modern technologies has led to a massive growth of high-resolution observational data in geosciences [2, 3]. For instance, in the petroleum industry, Permanent Reservoir Monitoring (PRM) system is the cutting-edge technology used to collect 4-dimensional (4D) seismic data. The frequent multiple vintages of 4D seismic result in huge datasets, with the size of total data often in the order of hundreds of millions, or even higher. Therefore, there is a high demand from the petroleum industry for efficient methods of big data analytics to extract, analyze and utilize the information from big 4D seismic data. Similar problems are also faced in many other fields that involve abundant data obtained through, for example, satellite remote sensing [4], medical imaging [5], geophysical surveys [6, 7], and so on. As a result, big (geophysical) data assimilation has become an important topic in practice.

In this study, we focus on seismic data assimilation problems (also known as seismic history matching problems in the petroleum industry). In the petroleum industry, seismic is one of the most important tools used for reservoir exploration, monitoring, characterization and management. Compared to conventional production data used in history matching, seismic data is less frequent in time, but much denser in space. Therefore, complementary to production data, seismic data provide valuable additional information for reservoir characterization.

The main contribution of the current study is to extend an ensemble-based seismic history matching (SHM) framework in [8] to handle 3D reservoir characterization problems, and such an extension will be an important step towards real field applications. Compared to similar existing frameworks in reservoir engineering community, our proposed SHM framework consists of some relatively new ingredients, in terms of the seismic data in use, wavelet multiresolution analysis for the chosen seismic data and related data noise estimation, and the use of recently developed iterative ensemble history matching algorithms.

The core of our proposed SHM framework resides in two aspects. One is to reduce the size of big data, while retaining sufficient information for data assimilation; and the other is to quantify data uncertainties in the course of data-size reduction. Data reduction is a natural (and often necessary) step in big data analytics, as can be found in many other fields like geosciences [9], business management [10], and social or geo-spatial network [11] described by complex network theory [12–16]. Meanwhile, quantifying the uncertainties of observational data is a key step towards successful data assimilation. Consequently, devising a workflow that achieves simultaneous data reduction and uncertainty quantification will render an efficient big data assimilation framework. In the sequel, we elaborate how such a framework can be established in the context of ensemble-based data assimilation.

In the data assimilation community, ensemble-based methods [17] are among the state-of-the-art assimilation algorithms. The first ensemble-based method, the ensemble Kalman filter (EnKF), was proposed in [18]. The EnKF can be considered as a Monte Carlo implementation of the classic Kalman filter (KF) [19]. For large-scale data assimilation problems, the EnKF is computationally much more efficient than the KF in terms of propagating forward in time model error covariance matrices (which are needed for model updates using observational data). In addition, the EnKF is very simple to implement, since it is derivative-free (e.g., no need to linearize the dynamical model and/or the observation system as in the extended Kalman filter [20]), and works even when the dynamical model and the observation system are black-box systems. Apart from the EnKF, the ensemble implementations of fixed-lag and fixed-interval smoothers are also developed in [21, 22]. These methods thus form a family of ensemble-based assimilation algorithms that can be deployed for different types of estimation (filtering or smoothing) problems. Due to the practical conveniences and good performances in real-world applications, in the past decades, ensemble-based methods have widely spread into various disciplines of geosciences, e.g., meteorology [23], oceanography [24], hydrology [25], reservoir engineering [26, 27], to name but a few.

A number of ensemble-based seismic history matching frameworks are already proposed in the literature to assimilate seismic data into reservoir models. For instance, [28–33] adopt the ensemble Kalman filter (EnKF) or a combination of the EnKF and ensemble Kalman smoother (EnKS), whereas [8, 34–36] employ the ensemble smoother with multiple data assimilation (ES-MDA), and regularized Levenburg-Marquardt (RLM) based iterative ensemble smoother (RLM-MAC, see [37]), respectively.

**Fig 1:**
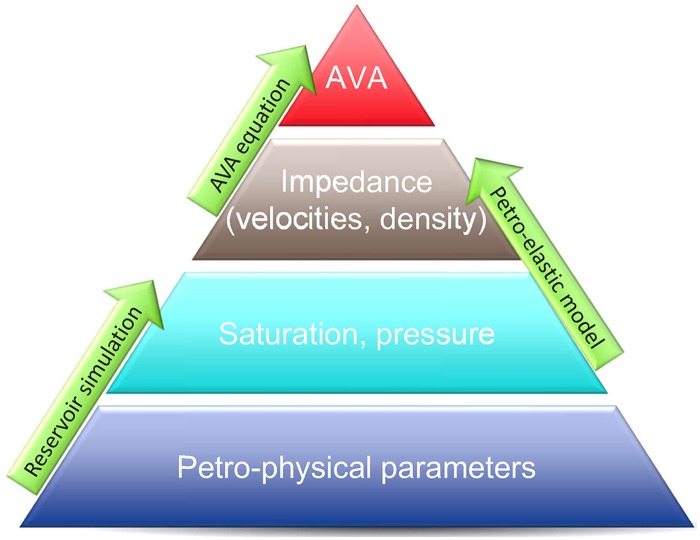
Some types of seismic data and their relation to reservoir petro-physical parameters.

There are different types of seismic data that one can use in SHM. Fig 1 provides an outline of the relation of some types of seismic data to reservoir petro-physical parameters (e.g., permeability and porosity) in forward simulations. As indicated there, using petro-physical parameters as the inputs to reservoir simulators, one generates fluid saturation and pressure fields. Through a petro-elastic model (PEM), one obtains acoustic and/or shear impedance (or equivalently, compressional and/or shear velocities (velocity)) and formation density based on fluid saturation and/or pressure, and porosity. Finally, amplitude versus angle (AVA) data are computed by plugging impedance (or velocities and density) into an AVA equation (e.g., Zoeppritz equation, see, for example, [38]).

To reduce the computational cost in forward simulations, many SHM studies use inverted seismic attributes that are obtained through seismic inversions. Such inverted properties can be, for instance, acoustic impedance (see, for example, [29, 32, 34, 35]) or fluid saturation fronts (see, for example, [28, 31, 33]). One issue in using inverted seismic attributes as the observational data is that, they may not provide uncertainty quantification for the observation errors, since inverted seismic attributes are often obtained using certain deterministic inversion algorithms. Quantifying the uncertainties in inverted seismic attributes is thus not a trivial issue [39, 40].

Since the volume of seismic data is typically huge, SHM often constitutes a “big data assimilation” problem. For ensemble-based history matching algorithms, a big data size may lead to certain numerical problems, e.g., ensemble collapse and high costs in computing and storing Kalman gain matrices [27, 34]. In addition, many history matching algorithms are developed for under-determined inverse problems (in which there are infinitely many solutions that could match the observational data exactly), whereas a big data size could make the inverse problem become over-determined instead (such that there is no solution that could match the observational data exactly). The presence of big data thus changes the nature of the inverse problem, and may affect the performance of the history matching algorithm in use, as demonstrated in [8].

**Fig 2:**
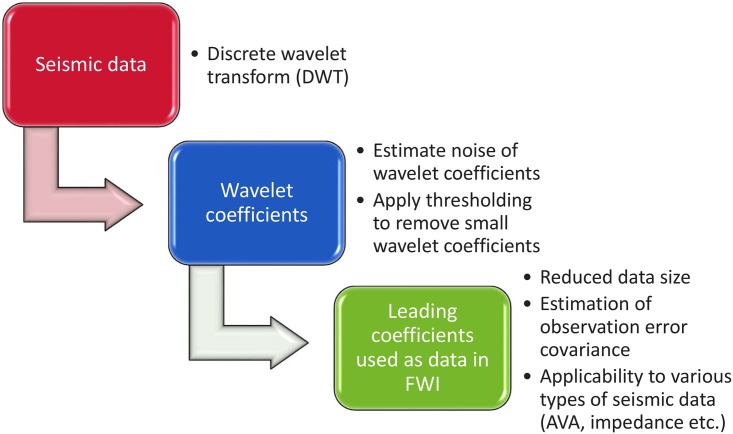
Workflow of wavelet-based sparse representation.

To avoid the extra uncertainties arising from a seismic inversion process, 2D AVA attributes are adopted as the seismic data in [8]. A wavelet-based sparse representation procedure is then adopted to transform the 2D datasets into the wavelet domain for data-size reduction and uncertainty quantification. Fig 2 explains the idea behind the wavelet-based sparse representation procedure. Given a set of 2D seismic data, one first applies a multilevel 2D discrete wavelet transform (DWT) to the data. 2D DWT is adopted there for the following two purposes: one is to reduce the size of seismic data by exploiting the sparse representation nature of wavelet basis functions, and the other is to exploit its capacity of noise estimation in the wavelet domain [41]. Based on the estimated standard deviation (STD) of the noise, one can construct the corresponding observation error covariance matrix that is needed in ensemble-based history matching algorithms.

For a chosen family of wavelet basis functions, seismic data are represented by the corresponding wavelet coefficients. When dealing with 2D data, 2D DWT is similar to singular value decomposition (SVD) applied to a matrix. In the latter case, the matrix is represented by the corresponding singular values in the space spanned by the products of associated left and right singular vectors. Likewise, in the 2D case, one can also draw similarities between wavelet-based sparse representation and truncated singular value decomposition (TSVD). Indeed, in 2D DWT, small wavelet coefficients are typically dominated by noise, whereas large coefficients mainly carry signal information [42]. Therefore, as demonstrated in [8], it is possible for one to use only a small subset of leading wavelet coefficients to capture the main features of the 2D signal, while significantly reducing the number of wavelet coefficients. We remark that TSVD-based sparse representation is not a suitable choice in the context of history matching, since the associated basis functions (i.e., products of left and right singular vectors) are data-dependent, meaning that in general it is not meaningful to compare and match the singular values of observed and simulated data.

Wavelet-based sparse representation involves suppressing noise components in the wavelet domain. To this end, one first estimates the STD of the noise in wavelet coefficients, and then computes a threshold value that depends on both the noise STD and data size. One can substantially reduce the data size by only keeping leading wavelet coefficients above the threshold, while setting those below the threshold value to zero. The leading wavelet coefficients are then taken as the (transformed) seismic data, and are history-matched using a certain algorithm. In this way, one can achieve data-size reduction and uncertainty quantification simultaneously.

In real field case studies, seismic data are typically 3D datasets. Therefore, in this work, we focus on extending the 2D sparse representation procedure used in [8] to 3D problems. In this regard, one may first decompose a 3D dataset into a collection of 2D maps, and then apply the 2D sparse representation procedure to each 2D map, similar to the approach used in [43]. By doing so, however, the spatial correlations along a certain dimension may not be efficiently coded into the wavelet domain, and this may reduce the efficiency of data compaction. As a result, we follow the ideas in [44, 45] and directly apply 3D DWT to 3D seismic datasets. To accommodate this 3D extension, the method of noise STD estimation in the wavelet domain is also extended accordingly, following the rationale behind wavelet denoising [42].

This work is organized as follows. First, we introduce three key components of the proposed SHM framework, which includes: (1) forward AVA simulation, (2) 3D DWT based sparse representation procedure, and (3) regularized Levenburg-Marquardt based iterative ensemble smoother. Then, we apply the proposed framework to the 3D Brugge benchmark case, and investigate the performance of the proposed framework in various situations. Finally, we draw conclusions based on the results of our investigation and discuss possible future work.

## The proposed framework

**Fig 3:**
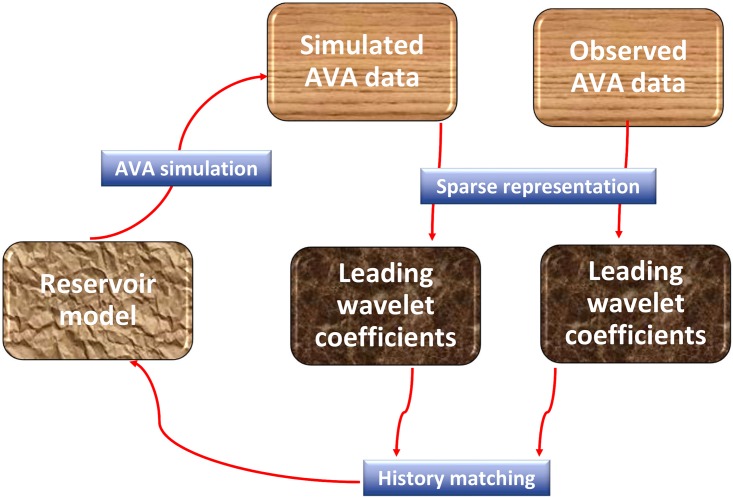
The proposed 4D seismic history matching framework.

The proposed framework consists of three key components (see Fig 3), namely, forward AVA simulation, sparse representation (in terms of leading wavelet coefficients) of both observed and simulated AVA data, and the history matching algorithm. It is expected that the proposed framework can also be extended to other types of seismic data, and more generally, geophysical data with spatial correlations.

### Forward AVA simulation

AVA data describes variations of seismic amplitudes with the change of distances between sources and receivers. Typically, the variations in AVA data can be attributed to the changes in lithology and/or fluid contents inside hydrocarbon reservoirs. As a result, the variations in AVA data are often used by geophysicists to estimate static and dynamical properties (e.g., porosity, pressure and fluid saturations) and/or their changes in hydrocarbon reservoirs [46–49]. In the context of SHM, the forward AVA simulation involves several steps as shown in Fig 1. First, pore pressure and fluid saturations are generated through reservoir flow simulation that takes petro-physical (e.g., permeability and porosity) and other parameters as the inputs. The generated pressure and saturation values are then used to calculate seismic parameters, such as P- and S-wave velocities and densities of reservoir and overburden formations, through a petro-elastic model (PEM). Finally, a certain AVA equation is adopted to compute the AVA attributes at different angles or offsets.

In practice, constructing a proper PEM is crucial to the success of SHM. To interpret the changes in seismic response over time, an in-depth understanding of rock and fluid properties is required [50]. A proper PEM is often initially built upon our basic understanding of the properties of reservoir formation, and then adjusted and calibrated to match (typically a limited number of) observational data from well logs and/or other sources [51]. In this study, since our focus is on validating the performance of the proposed SHM framework in a 3D problem, we assume that the PEM is perfect. Here, we use the soft sand model as the PEM [51]. The model assumes that, the cement is deposited away from the grain contacts. It further considers that, the initial framework of the uncemented sand rock is densely random pack of spherical grains with the porosity (denote by *ϕ* hereafter) around 36%, which is the maximum porosity value that the rock could have before suspension. For convenience of discussion later, we denote this value as the critical porosity (*ϕ*_*c*_) [52, 53]. The dry bulk modulus (*K*_*HM*_) and shear modulus (*μ*_*HM*_) at critical porosity can then be computed using the Hertz-Mindlin model [54] below

$$K_{HM}=\sqrt[{n}]{\frac{C_{p}^{2}{\left(1-\phi_{c}\right)}^{2}\mu_{s}^{2}}{18\pi^{2}{\left(1-\nu_{s}\right)}^{2}}P_{eff}},$$ (1)

and

$$\begin{array}{c} \mu_{HM}=\frac{5-4\nu_{s}}{5(2-\nu_{s})}\sqrt[{n}]{\frac{3C_{p}^{2}{\left(1-\phi_{c}\right)}^{2}\mu_{s}^{2}}{2\pi^{2}{\left(1-\nu_{s}\right)}^{2}}P_{eff}}\;, \end{array}$$ (2)

where *μ*_*s*_, *ν*_*s*_, *P*_*eff*_ are grain shear modulus, Poisson’s ratio, and effective stress, respectively. The coordination number *C*_*p*_ denotes the average number of contacts per sphere, and *n* is the degree of root. Here, *C*_*p*_ and *n* are set to 9 and 3, respectively.

To find the effective dry moduli for a porosity value less than *ϕ*_*c*_, the modified Lower Hashin-Shtrikman (MLHS) bound can be used [51]. The MLHS connects two end points in the elastic modulus-porosity plane. One end point, (*K*_*HM*_, *μ*_*HM*_), corresponds to critical porosity *ϕ*_*c*_. The other end point corresponds to zero porosity, taking the moduli of the solid phase, i.e. quartz mineral (*K*_*s*_, *μ*_*s*_). For a porosity value *ϕ* between zero and *ϕ*_*c*_, the lower bound for dry rock effective bulk (*K*_*d*_) and shear (*G*_*d*_) moduli can be expressed as

$$\begin{array}{c} K_{d}={\left[\frac{\frac{\phi }{\phi_{c}}}{K_{HM}+\frac{4}{3}\mu_{HM}}+\frac{\frac{1-\phi }{\phi_{c}}}{K_{s}+\frac{4}{3}\mu_{HM}}\right]}^{-1}-\frac{4}{3}\mu_{HM} \end{array}$$ (3)

and

$$\begin{array}{c} G_{d}={\left[\frac{\frac{\phi }{\phi_{c}}}{\mu_{HM}+\frac{\mu_{HM}}{6}Z}+\frac{\frac{1-\phi }{\phi_{c}}}{\mu_{s}+\frac{\mu_{HM}}{6}Z}\right]}^{-1}-\frac{\mu_{HM}}{6}Z, \end{array}$$ (4)

respectively, where *K*_*s*_ is solid/mineral bulk modulus and *Z* = (9*K*_*HM*_ + 8*μ*_*HM*_)/(*K*_*HM*_ + 2*μ*_*HM*_).

Further, the saturation effect is incorporated using the Gassmann model [55]. The saturated bulk modulus (*K*_*sat*_) and shear modulus (*μ*_*sat*_) can be expressed as

$$\begin{array}{c} K_{sat}=K_{d}+\frac{{\left(1-\frac{K_{d}}{K_{s}}\right)}^{2}}{\frac{\phi }{K_{f}}+\frac{1-\phi }{K_{s}}-\frac{K_{d}}{K_{s}^{2}}}\;, \end{array}$$ (5)

and

$$\begin{array}{c} \mu_{sat}=\mu_{d}\;, \end{array}$$ (6)

respectively, where *K*_*f*_ is the effective fluid bulk modulus, and is estimated using the Reuss average [56]. For an oil-water mixture (as is the case in the Brugge field), *K*_*f*_ is given by

$$\begin{array}{c} K_{f}={\left(\frac{S_{w}}{K_{w}}+\frac{S_{o}}{K_{o}}\right)}^{-1}\;, \end{array}$$ (7)

where *K*_*w*_, *K*_*o*_, *S*_*w*_ and *S*_*o*_ are bulk modulus of water/brine, bulk modulus of oil, saturation of water/brine and saturation of oil, respectively.

Further, the saturated density [51] can be written as (for the water-oil mixture)

$$\begin{array}{c} \rho_{sat}=\left(1-\phi \right)\rho_{m}+\phi S_{w}\rho_{w}+\phi S_{o}\rho_{o}\;, \end{array}$$ (8)

where *ρ*_*sat*_, *ρ*_*m*_, *ρ*_*w*_ and *ρ*_*o*_ are saturated density of rock, mineral density, water/brine density and oil density, respectively.

Using the above equations, we can obtain P- and S-wave velocities given by [51]

$$\begin{array}{c} V_{P}=\sqrt{\frac{K_{sat}+\frac{4}{3}\mu_{sat}}{\rho_{sat}}}\;, \end{array}$$ (9)

and

$$\begin{array}{c} V_{S}=\sqrt{\frac{\mu_{sat}}{\rho_{sat}}}\;, \end{array}$$ (10)

where *V*_*P*_ and *V*_*S*_ represent P- and S-wave velocities, respectively.

After seismic parameters are generated by plugging reservoir parameters into the PEM, we can then simulate seismogram based on these seismic parameters. First, the Zoeppritz equation is used to calculate the reflection coefficient at an interface between two layers. For multi-layer cases, we need to calculate reflectivity series as a function of two-way travel time (see, for example, [47, 51]). Here, travel time is computed from the P-wave velocity and vertical thickness of each grid block. We then convolve the reflectivity series with a Ricker wavelet of 45 Hz dominant frequency to obtain the desired seismic AVA data. In the experiments later, we generate AVA data at angles of 10° and 20°, and call them near- and far-offset attributes, respectively. We choose not to further convert theses AVA data into other attributes (e.g., intercept and gradient) to avoid introducing extra nonlinearities in the course of attribute conversion.

### Sparse representation and noise estimation in the wavelet domain

Let $m^{ref}\in R^{m}$ denote the reference reservoir model. In the current study, we consider 3D AVA attributes (near- and far-offset traces) in the form of *p*_1_ × *p*_2_ × *p*_3_ arrays (tensors), where *p*_1_, *p*_2_ and *p*_3_ represent the numbers of inline, cross-line and time slices in seismic surveys, respectively. Accordingly, let $g:R^{m}\to R^{p_{1}\times p_{2}\times p_{3}}$ be the forward simulator of AVA attributes. The observed AVA attributes **d**^*o*^ are supposed to be the forward simulation **g**(**m**^*ref*^) with respect to the reference model, plus certain additive observation errors ***ϵ***, that is,

$$\begin{array}{c} d^{o}=g\left(m^{ref}\right)+\epsilon \;. \end{array}$$ (11)

For ease of discussion below, suppose at the moment that all the tensors in Eq (11), i.e., **d**^*o*^, **g**(**m**^*ref*^) and ***ϵ***, are reshaped into vectors with *p*_1_ × *p*_2_ × *p*_3_ elements. Throughout this study, we assume that, for a given AVA attribute, the elements of ***ϵ*** are independently and identically distributed (i.i.d) Gaussian white noise, with zero mean but unknown variance *σ*^2^, where *σ* will be estimated through wavelet multiresolution analysis below. More generally, one may also assume that ***ϵ*** follows a Gaussian distribution with zero mean and covariance **R**, where **R** is not known a priori in practice. In this case, one may still apply a wavelet-denoising-based method to estimate the noise STD in the wavelet domain, see, for example, [40].

As shown in Fig 2, wavelet-based sparse representation involves the following steps: (I) Apply DWT to seismic data; (II) Estimate noise STD of wavelet coefficients; and (III) Compute a threshold value that depends on both noise STD and data size, and do thresholding accordingly. Sparse representation (either wavelet-based or not) is also used for other purposes in petroleum engineering. For instance, it is adopted in [57–64] for re-parametrization of reservoir models, rather than size reduction of observational data. [57] also used wavelet-based sparse representation to represent time-lapse saturation maps. However, [57] does not consider the estimation of noise STD of wavelet coefficients, which is an issue to be addressed below.

To handle 3D seismic datasets, we need to extend the 2D sparse representation procedure used in [8] to 3D problems. To this end, instead of applying 2D DWT to a collection of 2D seismic maps as suggested in [43], we directly apply 3D DWT to the 3D datasets, in light of the works [44, 45].

**Fig 4:**
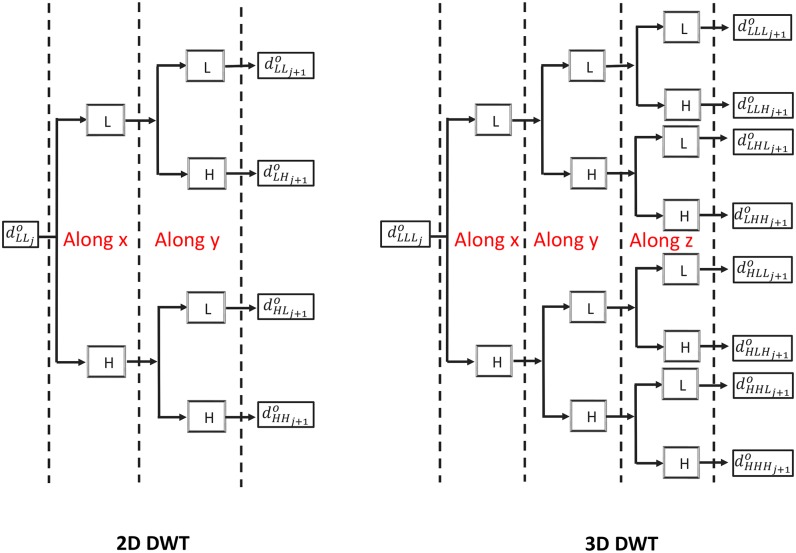
Single level decompositions in 2D (left) and 3D (right) decimated discrete wavelet transforms.

For brevity, an introduction to wavelet theory is omitted in this work. Interested readers are referred to, for example, [65]. Instead, we focus more on explaining how wavelet transform is used for sparse representation and noise estimation in the wavelet domain. Before we proceed, let us first discuss the differences between 2D and 3D DWTs. Fig 4 illustrates single level decompositions in 2D (left) and 3D (right) DWTs, respectively. For convenience of discussion, we label the dataset dimensions by *x* and *y* in 2D DWT, and *x*, *y* and *z* in 3D DWT. Without loss of generality, we assume that the data is $d_{LLL_{j}}^{o}$ in a 3D DWT ($d_{LL_{j}}^{o}$ in a 2D DWT), which can be either the original observation **d**^*o*^ (when *j* = 0) or the partial data recovered using the wavelet coefficients of the sub-band *LLL*_*j*_ in 3D DWT (*LL*_*j*_ in 2D DWT) when *j* > 0. Conventionally, a multiple dimensional DWT is implemented by sequentially applying 1D DWTs along different directions [42]. In each 1D DWT, there are both low- (L) and high-pass (H) filter banks, and the transform results in one “L” and one “H” sub-band of wavelet coefficients (intuitively, the “H” sub-band corresponds to high frequency components in the wavelet domain, while the “L” sub-band to low frequency ones). Therefore, given the dataset dimension *dim*, a single level wavelet decomposition would result in 2^*dim*^ sub-bands at the next level, e.g., four (*dim* = 2) for 2D DWT and eight (*dim* = 3) for 3D DWT. For convenience, one can tag these sub-bands by a combination of “H” and “L” labels. Here, let us assume *j* = 0, such that Fig 4 corresponds to the first level 3D (or 2D) wavelet transform. For 3D DWT, after a single level DWT, there are 8 sub-bands in the wavelet domain, which are labeled as *LLL*_1_, *LLH*_1_, *LHL*_1_, *LHH*_1_, *HLL*_1_, *HLH*_1_, *HHL*_1_ and *HHH*_1_, respectively. The sub-band *LLL*_1_ (*HHH*_1_) results only from low-pass (high-pass) filters, while the others from mixtures of low- and high-pass ones. One can continue the 3D DWT to the next level by applying the transform to the data $d_{LLL_{1}}^{o}$ that corresponds to the sub-band *LLL*_1_. This leads to a set of new sub-bands of wavelet coefficients (labelled as *LLL*_2_, *LLH*_2_, *LHL*_2_, *LHH*_2_, *HLL*_2_, *HLH*_2_, *HHL*_2_ and *HHH*_2_, respectively), and so on. The situation in 2D DWT is similar, but with a less number of sub-bands (labeled by *LL*_*j*_, *LH*_*j*_, *HL*_*j*_ and *HH*_*j*_ in general) at each level of wavelet decomposition.

For our purpose in the current study, we focus on 3D DWT in the sequel. In this case, since *HHH*_1_ corresponds to the high frequency (typically noise) components of the original data **d**^*o*^, it can be used to infer noise STD in the wavelet domain, similar to the idea used in [8]. Specifically, let W and T denote orthogonal wavelet transform and thresholding operators, respectively; ${\tilde{d}}^{o}=W\left(d^{o}\right)$ stands for the whole set of wavelet coefficients corresponding to the original data **d**^*o*^, and ${\tilde{d}}_{HHH_{1}}^{o}$ for the wavelet coefficients in the sub-band *HHH*_1_. After DWT and thresholding, the effective observation system becomes

$$\begin{array}{c} T\circ W\left(d^{o}\right)=T\circ (W\circ g\left(m^{ref}\right)+W\left(\epsilon \right))\;. \end{array}$$ (12)

As will be discussed below, for leading wavelet coefficients (those above the threshold value), T is an identity operator such that it does not modify the values of leading wavelet coefficients. The reason for us to require orthogonal W is as follows: If W is orthogonal, then the wavelet transform preserves the energy of Gaussian white noise (e.g., the Euclidean norm of the noise term ***ϵ***). In addition, like the power spectral distribution of white noise in frequency domain, the noise energy in the wavelet domain is uniformly distributed among all wavelet coefficients [42]. This implies that, if one can estimate the noise STD *σ* of small wavelet coefficients (e.g., those in *HHH*_1_), then this estimation can also be used to infer the noise STD of leading wavelet coefficients used in history matching. Similar to our previous study [8], the noise STD *σ* is estimated using the median absolute deviation (MAD) estimator [41]:

$$\begin{array}{c} \sigma =\frac{\text{median}(\text{abs}\left({\tilde{d}}_{HHH_{1}}^{o}\right))}{0.6745}\;, \end{array}$$ (13)

where abs(•) is an element-wise operator, and takes the absolute value of an input quantity.

After estimating *σ* in an *n*-level wavelet decomposition, we apply hard thresholding and select leading wavelet coefficients on the element-by-element basis, in a way such that

$$\begin{array}{c} T\left({\tilde{d}}_{i}^{o}\right)=\{\begin{array}{cc} 0 & \;\text{if}\;|{\tilde{d}}_{i}^{o}|<\lambda \;,\\ {\tilde{d}}_{i}^{o} & \;\text{otherwise}\;, \end{array} \end{array}$$ (14)

where, without loss of generality, the scalar ${\tilde{d}}_{i}^{o}\in {\tilde{d}}^{o}$ represents an individual wavelet coefficient, and λ is a certain threshold value to be computed later. Eq (14) means that, for leading wavelet coefficients above (or equal to) the threshold λ, their values are not changed, whereas for those below λ, they are set to zero. Note that in [8], hard thresholding is not applied to the coarsest sub-band (i.e., the *LL*_*n*_/*LLL*_*n*_ sub-band for an *n*-level 2D/3D DWT) in light of the fact that the wavelet coefficients in this sub-band correspond to low-frequency components, which are typically dominated by the signal. As a result, applying thresholding to this sub-band may lead to certain loss of signal information in history matching. However, for an AVA attribute in this study, we have observed that its corresponding *LLL*_*n*_ sub-band may contain a large amount of wavelet coefficients (e.g., in the order of 10^4^). To have the flexibility of efficiently reducing the data size, we lift the restriction such that thresholding can also be applied to the sub-band *LLL*_*n*_. By doing so, however, some extra (relatively small) low-frequency wavelet coefficients are discarded, such that one may end up with more loss of data information content.

In [8], the threshold value λ is computed using the universal rule [66]

$$\begin{array}{c} \lambda =\sqrt{2\;\text{ln}(\#{\tilde{d}}^{o})}\;\sigma \;, \end{array}$$ (15)

with $\#{\tilde{d}}^{o}$ being the number of elements in ${\tilde{d}}^{o}$. The rationale behind the universal rule is as follows: suppose that all the elements of a set ${\tilde{d}}^{o}$ (e.g. wavelet coefficients) are noise samples drawn from the Gaussian distribution *N*(0, *σ*^2^), then the probability $pr(\text{max}\left(abs\left({\tilde{d}}^{o}\right)\right)<\lambda )\to 1$ as the number of elements of ${\tilde{d}}^{o}$ tends to ∞ [65]. Therefore, if an element of ${\tilde{d}}^{o}$ is larger than the threshold λ, then it is more likely to be a signal component, than to be a noise component. This viewpoint thus underpins the thresholding strategy in Eq (14).

In the current work, when using Eq (15) to select the threshold value, it is found that the resulting number of leading wavelet coefficients may still be very large. As a result, in the experiments later, we select the threshold value according to

$$\begin{array}{c} \lambda =c\;\sqrt{2\;\text{ln}(\#d^{o})}\;\sigma \;, \end{array}$$ (16)

where *c* > 0 is a positive scalar, and the larger the value of *c*, the less the number of leading wavelet coefficients. Therefore, the scalar *c* can be used to control the total number of leading wavelet coefficients.

Combining Eqs (12)–(16), the effective observation system in history matching becomes

$$\begin{array}{c} {\tilde{d}}^{o}=W\circ g\left(m^{ref}\right)+W\left(\epsilon \right)\;,\;\text{for}\;{\tilde{d}}^{o}\geq \lambda \;, \end{array}$$ (17)

where now ${\tilde{d}}^{o}$ is a vector containing all selected leading wavelet coefficients, and W(ϵ) the corresponding noise component in the wavelet domain, with zero mean and covariance $C_{{\tilde{d}}^{o}}=\sigma^{2}I$ (here **I** is the identity matrix with a suitable dimension).

**Fig 5:**
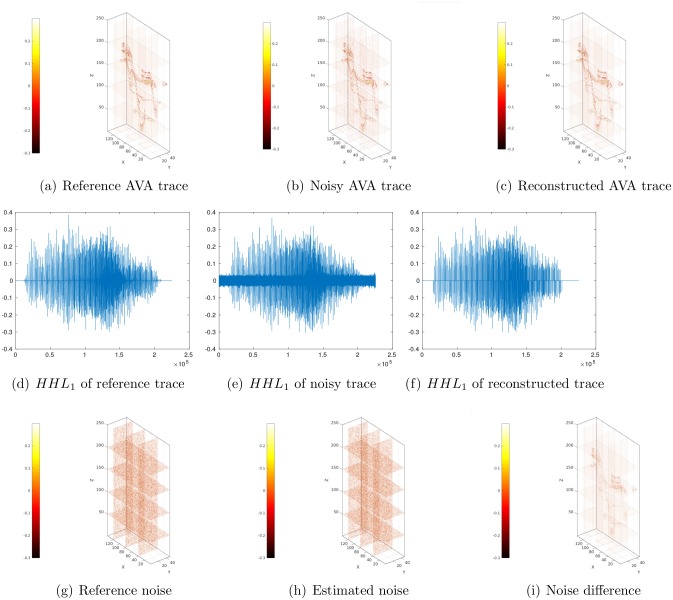
Illustration of sparse representation of a 3D AVA far-offset trace using slices at *X* = 40, 80, 120 and at *Z* = 50, 100, 150, 200, respectively. (a) Reference AVA trace; (b) Noisy AVA trace obtained by adding Gaussian white noise (noise level = 30%) to the reference data; (c) Reconstructed AVA trace obtained by first conducting a 3D DWT on the noisy data, then applying hard thresholding (using the universal threshold value) to wavelet coefficients, and finally reconstructing the data using an inverse 3D DWT based on the modified wavelet coefficients; (d) Wavelet sub-band *HHL*_1_ corresponding to the reference AVA data; (e) Wavelet sub-band *HHL*_1_ corresponding to the noisy AVA data; (f) Wavelet sub-band *HHL*_1_ corresponding to the reconstructed AVA data; (g) Reference noise, defined as noisy AVA data minus reference AVA data; (h) Estimated noise, defined as noisy AVA data minus reconstructed AVA data; (i) Noise difference, defined as estimated noise minus reference noise. All 3D plots are created using the package *Sliceomatic (version 1.1) from MATLAB Central (File ID: #764).*

We use an example to illustrate the performance of sparse representation and noise estimation in 3D DWT. In this example, we first generate a reference AVA far-offset trace using the forward AVA simulator. The dimension of this trace is 139 × 48 × 251, therefore the data size is 1, 674, 672. Fig 5A plots slices of the AVA trace at *X* = 40, 80, 120, and *Z* = 50, 100, 150 and 200, respectively. We then add Gaussian white noise to obtain the noisy AVA trace, with the noise level being 30% (we also tested the performance of Eq (13) in estimating noise STD with different noise levels, e.g, 0% and 10%, and obtained results similar to what are presented below). Here, noise level is defined as:

$$\begin{array}{c} \text{Noise}\;\text{level}\;=\frac{\text{variance}\;\text{of}\;\text{noise}}{\text{variance}\;\text{of}\;\text{pure}\;\text{signal}}\;. \end{array}$$ (18)

Fig 5B shows slides of the noisy AVA trace at the same locations as in Fig 5A.

We apply a three-level 3D DWT to the noisy data using Daubechies wavelets with two vanishing moments [65], and use hard thresholding combined with the universal rule (Eqs (13)–(15)) to select leading wavelet coefficients. After thresholding, the number of leading wavelet coefficients reduces to 33, 123, only 2% of the original data size. On the other hand, by applying Eq (13), the estimated noise STD is 0.0105, and it is very close to the true noise STD 0.0104. By applying an inverse 3D DWT to leading wavelet coefficients, we obtain the reconstructed AVA trace (Fig 5C plots slices of this trace at the same places as Fig 5A and 5B). Comparing Fig 5A–5C, one can see that, using leading wavelet coefficients that amounts to only 2% of the original data size, the slices of reconstructed AVA trace well capture the main features in the corresponding slices of the reference AVA data.

Fig 5D–5F show wavelet coefficients in the sub-bands *HHL*_1_ of the reference, noisy and reconstructed AVA traces, respectively. From these figures, one can see that, after applying thresholding to wavelet coefficients of noisy data (Fig 5E), the modified coefficients (Fig 5F) preserve those with large amplitudes in the reference case (Fig 5D). In general, the modified coefficients appear similar to those of the reference case, whereas certain small coefficients of the reference case are suppressed due to thresholding.

Finally, Fig 5G–5I depict slices of reference and estimated noise, and their difference, respectively, at the same places as in Fig 5A. Here, reference noise is defined as noisy AVA data (Fig 5B) minus reference AVA data (Fig 5A), estimated noise as noisy AVA data minus reconstructed AVA data (Fig 5C), and noise difference as estimated noise minus reference noise. The estimated noise appears very similar to the reference noise, although there are also certain differences according to Fig 5I. This might be largely due to the fact that some small wavelet coefficients of the reference data are smeared out after thresholding, as aforementioned.

### The ensemble history matching algorithm

We adopt the RLM-MAC algorithm [37] in history matching. Without loss of generality, let **d**^*o*^ denote *p*-dimensional observations in history matching, which stands for values in the ordinary data space (e.g., 3D AVA attributes by reshaping 3D arrays into vectors), or their sparse representations in the transform domain (e.g., leading wavelet coefficients in the wavelet domain). The observations **d**^*o*^ are contaminated by Gaussian noise with zero mean and covariance **C**_*d*_ (denoted by **d**^*o*^ ∼ *N*(**0**, **C**_*d*_)). Also denote by **g** the forward simulator that generates simulated observations **d** ≡ **g**(**m**) given an *m*-dimensional reservoir model **m**.

In the RLM-MAC algorithm, let $M^{i}\equiv {\left\{m_{j}^{i}\right\}}_{j=1}^{N_{e}}$ be an ensemble of *N*_*e*_ reservoir models obtained at the *i*th iteration step, based on which we can construct two square root matrices used in the RLM-MAC algorithm. One of the matrices is in the form of

$$\begin{array}{cc} S_{m}^{i}=\frac{1}{\sqrt{N_{e}-1}}\left[m_{1}^{i}-{\bar{m}}^{i},\cdots ,m_{N_{e}}^{i}-{\bar{m}}^{i}\right]; & {\bar{m}}^{i}=\frac{1}{N_{e}}\sum_{j=1}^{N_{e}}m_{j}^{i}, \end{array}$$ (19)

and is called *model square root matrix*, in the sense that $C_{m}^{i}\equiv S_{m}^{i}{\left(S_{m}^{i}\right)}^{T}$ equals the sample covariance matrix of the ensemble **M**^*i*^. The other, defined as

$$S_{d}^{i}=\frac{1}{\sqrt{N_{e}-1}}\left[g(m_{1}^{i})-g({\bar{m}}^{i}),\cdots ,g(m_{N_{e}}^{i})-g({\bar{m}}^{i})\right],$$ (20)

is called *data square root matrix* for a similar reason.

The RLM-MAC algorithm updates **M**^*i*^ to a new ensemble $M^{i+1}\equiv {\left\{m_{j}^{i+1}\right\}}_{j=1}^{N_{e}}$ by solving the following minimum-average-cost problem

$$\begin{array}{cc} \underset{{\left\{m_{j}^{i+1}\right\}}_{j=1}^{N_{e}}}{\text{argmin}}\frac{1}{N_{e}}\overset{N_{e}}{\underset{j=1}{\Sigma }} & [{\left(d_{j}^{o}-g\left(m_{j}^{i+1}\right)\right)}^{T}C_{d}^{-1}\left(d_{j}^{o}-g\left(m_{j}^{i+1}\right)\right)+\\ & \gamma^{i}{\left(m_{j}^{i+1}-m_{j}^{i}\right)}^{T}{\left(C_{m}^{i}\right)}^{-1}\left(m_{j}^{i+1}-m_{j}^{i}\right)], \end{array}$$ (21)

where $d_{j}^{o}$ (*j* = 1, 2, ⋯, *N*_*e*_) are perturbed observations generated by drawing *N*_*e*_ samples from the Gaussian distribution *N*(**d**^*o*^, **C**_*d*_), and *γ*^*i*^ a positive scalar that can be used to control the step size of an iteration step, and is automatically chosen using a procedure similar to back-tracking line search [37]. Through linearization, the MAC problem is approximately solved through the following iteration:

$$m_{j}^{i+1}=m_{j}^{i}+S_{m}^{i}{\left(S_{d}^{i}\right)}^{T}{\left(S_{d}^{i}{\left(S_{d}^{i}\right)}^{T}+\gamma^{i}C_{d}\right)}^{-1}\left(d_{j}^{o}-g\left(m_{j}^{i}\right)\right),\;\text{for}\;j=1,\cdots ,N_{e}.$$ (22)

The stopping criteria have substantial impact on the performance of an iterative inversion algorithm [67]. [37] mainly used the following two stopping conditions for the purpose of run-time control:

(C1) RLM-MAC stops if it reaches a maximum number of iteration steps;
(C2) RLM-MAC stops if the relative change of average data mismatch over two consecutive iteration steps is less than a certain value.

For all the experiments later, we set the maximum number of iterations to 20, and the limit of the relative change to 0.01%.

Let

$$\Xi^{i}\equiv \frac{1}{N_{e}}\sum_{j=1}^{N_{e}}\left[{\left(d_{j}^{o}-g\left(m_{j}^{i}\right)\right)}^{T}C_{d}^{-1}\left(d_{j}^{o}-g\left(m_{j}^{i}\right)\right)\right]$$ (23)

be the average (normalized) data mismatch with respect to the ensemble **M**^*i*^. Following Proposition 6.3 of [67], a third stopping condition is introduced and implemented in [8]. Concretely, we also stop the iteration in Eq (22) when

$$\Xi^{i}<4p$$ (24)

for the first time, where the factor 4 is a critical value below which the iteration process starts to transit from convergence to divergence [67]. Numerical results in [8] indicate that, in certain circumstances, equipping RLM-MAC with the extra stopping condition (24) may substantially improve its performance in history matching. Readers are referred to [8] for more details. In the current study, however, the impact of the stopping criterion (24) is not as substantial as that in [8]. Nevertheless, we prefer to keep this stopping criterion as an extra safeguard procedure.

## Numerical results in the Brugge benchmark case

We demonstrate the performance of the proposed workflow through a 3D Brugge benchmark case study. Table 1 summarizes the key information of the experimental settings. Readers are referred to [68] for more information of the benchmark case study.

**Table 1:** Summary of experimental settings in the Brugge benchmark case study.

Model dimension	139 × 48 × 9 (60048 gridblocks), with 44550 out of 60048 being active cells
Parameters to estimate	PORO, PERMX, PERMY, PERMZ. Total number is 4 × 44550 = 178200
Gridblock size	Irregular. Average Δ*X* ≈ 93*m*, Δ*Y* ≈ 91*m*, and average Δ*Z* ≈ 5*m*
Reservoir simulator	ECLIPSE 100 (control mode LRAT)
Number of wells	10 injectors and 20 producers
Production period	3647.5 days (with 20 report times)
Production data	Production wells: BHP, OPR and WCT; Injection wells: BHP. Total number: 20 × 70 = 1400
Seismic survey time	Base: day 1; Monitor (1st): day 991; Monitor (2nd): day 2999
4D seismic data	AVA data from near- and far- offsets at three survey times. Total number: ∼ 7.02 M
DWT (seismic)	Three-level decomposition using 3D Daubechies wavelets with two vanishing moments
Thresholding	Hard thresholding based on Eqs (14) and (16)
History matching method	iES (RLM-MAC) with an ensemble of 103 reservoir models

The Brugge field model has 9 layers, and each layer consists of 139 × 48 gridblocks. The total number of gridblocks is 60048, whereas among them 44550 are active. The data set of the original benchmark case study does not contain AVA attributes, therefore we generate synthetic seismic data in the following way: The benchmark case contains an initial ensemble of 104 members. We randomly pick one of them as the reference model (which turned out to be the member “FN-SS-KP-1-92”), and use the rest 103 members as the initial ensemble in this study. The model variables to be estimated include porosity (PORO) and permeability (PERMX, PERMY, PERMZ) at all active gridblocks. Consequently, the total number of parameters in estimation is 178200.

There are 20 producers and 10 water injectors in the reference model, and they are controlled by the liquid rate (LRAT) target. The production period is 10 years, and in history matching we use production data at 20 report times. The production data consist of oil production rates (WOPR) and water cuts (WWCT) at 20 producers, and bottom hole pressures (WBHP) at all 30 wells. Therefore the total number of production data is 1400. Gaussian white noise is added to production data of the reference model. For WOPR and WWCT data, their noise STD are taken as the maximum values between 10% of their magnitudes and 10^−6^ (the latter is used to prevent the numerical issue of division by zero), whereas for WBHP data, the noise STD is 1 bar.

In the experiments, there are three seismic surveys taking place on day 1 (base), day 991 (1st monitor), and day 2999 (2nd monitor), respectively. At each survey, we apply forward AVA simulation described in the previous section to the static (porosity) and dynamic (pressure and saturation) variables of the reference model, and generate AVA attributes at two different angles: 10° (near-offset) and 20° (far-offset). Each AVA attribute is a 3D (139 × 48 × 176) cube, and consists of around 1.17 × 10^6^ elements. Therefore the total number of 4D seismic data is around 3 × 2 × 1.17 × 10^6^ = 7.02 × 10^6^. For convenience of discussion later, we label the dimensions of the 3D cubes by *X*, *Y* and *Z*, respectively, such that *X* = 1, 2, ⋯, 139, *Y* = 1, 2, ⋯, 48 and *Z* = 1, 2, ⋯, 176. In history matching, we add Gaussian white noise to each reference AVA attribute, with the noise level being 30%. Here we do not assume the noise STD is known. Instead, we first apply three-level 3D DWT to each AVA attribute using Daubechies wavelets with two vanishing moments, and then use Eq (13) to estimate noise STD in the wavelet domain.

In what follows, we consider three history matching scenarios that involve: (S1) production data only; (S2) 4D seismic data only; and (S3) both production and 4D seismic data. Because of the huge volumes of AVA attributes, in scenarios (S2) and (S3), we cannot directly use the 4D seismic data in the original data space (although this was done in the 2D problem of [8]). Indeed, in one of our experiments that attempted to use the original seismic dataset, we encountered the problem of running out of computer memory. Therefore, in the current work, to examine the impact of data size on the performance of SHM, in each scenario (S2 or S3), we consider two cases that have different numbers of leading wavelet coefficients. This is achieved by letting the scalar *c* of Eq (16) be 1 and 5, respectively. In contrast, for production data in (S1) and (S3), no wavelet transform is applied as the data size is already small.

### Results of scenario S1 (using production data only)

**Fig 6:**
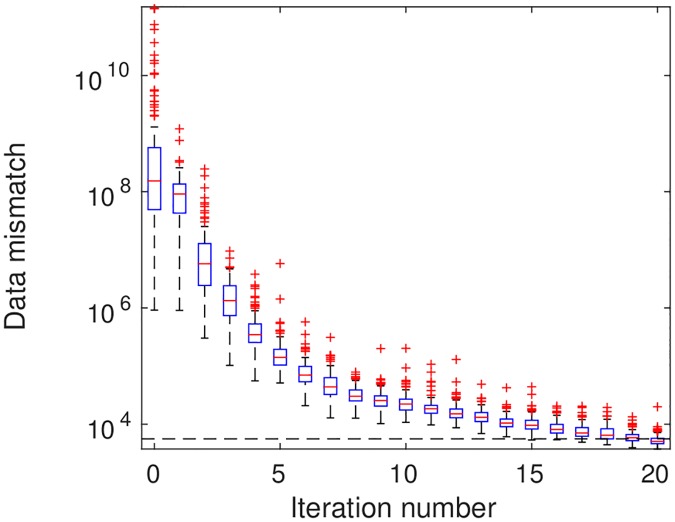
Boxplots of production data mismatch as a function of iteration step (scenario S1). The horizontal dashed line indicates the threshold value (4 × 1400 = 5600) for the stopping criterion (24). For visualization, the vertical axis is in the logarithmic scale. In each box plot, the horizontal line (in red) inside the box denotes the median; the top and bottom of the box represent the 75th and 25th percentiles, respectively; the whiskers indicate the ranges beyond which the data are considered outliers, and the whiskers positions are determined using the default setting of MATLAB R2015b, while the outliers themselves are plotted individually as plus signs (in red).

Fig 6 shows the boxplots of data mismatch as a function of iteration step. The average data mismatch of the initial ensemble (iteration 0) is around 5.65 × 10^9^. After 20 iteration steps, the average data mismatch is reduced to 5431.97, lower than the threshold value 4 × 1400 = 5600 in (24) for the first time. In this particular case, the stopping step selected according to the criterion (24) coincides with the maximum number of iteration steps. Therefore, we take the ensemble at the 20th iteration step as the final estimation.

In this synthetic study, the reference reservoir model is known. As a result, we use root mean squared error (RMSE) in the sequel to measure the *ℓ*_2_-distance (up to a factor) between an estimated model and the reference one. More specifically, let **v**^*tr*^ be the ℓ-dimensional reference property, and $\hat{v}$ an estimation, then the RMSE *e*_**v**_ of $\hat{v}$ with respect to the reference **v**^*tr*^ is defined by

$$e_{v}=\frac{\|\hat{v}-v^{tr}{\|}_{2}}{\sqrt{\ell }},$$ (25)

where ‖•‖_2_ denotes the *ℓ*_2_ norm.

**Fig 7:**
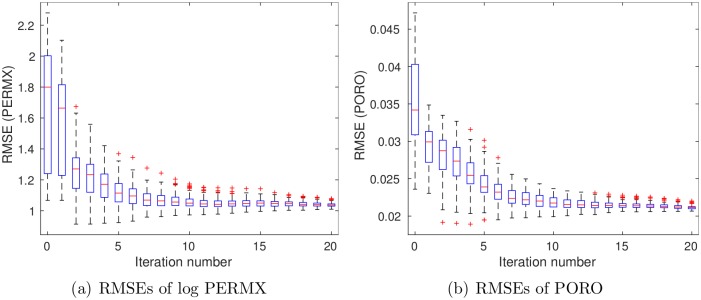
Boxplots of RMSEs of (a) log PERMX and (b) PORO as functions of iteration step (scenario S1).

For brevity, Fig 7 reports the boxplots of RMSEs in estimating PERMX (in the natural log scale) and PORO, at different iteration steps, whereas the results for PERMY and PERMZ are similar to that for PERMX. As can be seen in Fig 7, the average RMSEs of both log PERMX and PORO tend to reduce as the number of iteration steps increases.

**Fig 8:**
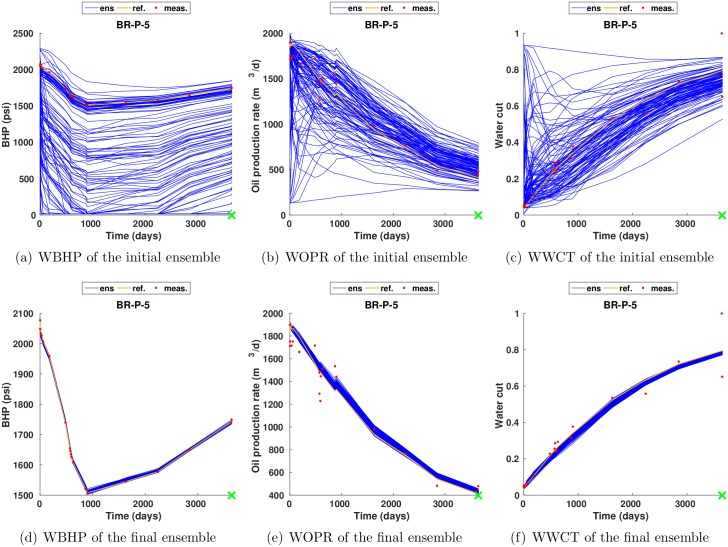
Profiles of WBHP, WOPR and WWCT of the initial (1st row) and final (2nd row) ensembles at the producer BR-P-5 (scenario S1). The production data of the reference model are plotted as orange curves, the observed production data at 20 report times as red dots, and the simulated production data of initial and final ensembles as blue curves.

Fig 8 shows the profiles of WBHP, WOPR and WWCT of the initial (1st row) and final (2nd row) ensemble at the producer BR-P-5. It is evident that, through history matching, the final ensemble matches the production data better than the initial one, and this is consistent with the results in Fig 6.

**Fig 9:**
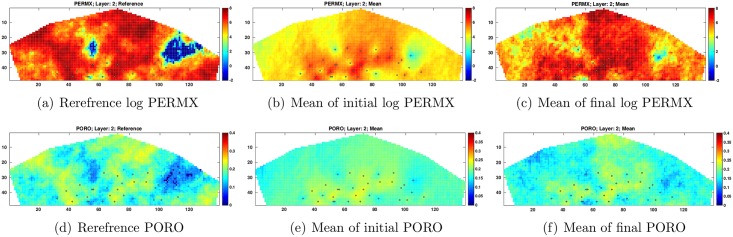
Log PERMX (top row) and PORO (bottom row) of the reference reservoir model (1st column) and the means of the initial (2nd column) and final (3rd column) ensembles at Layer 2 (scenario S1). The black dots in the figures represent the locations of injection and production wells (top view).

For illustration, Fig 9 presents the reference log PERMX and PORO at layer 2 (1st column), the mean of log PERMX and PORO at layer 2 from the initial ensemble (2nd column), and the mean of log PERMX and PORO at layer 2 from the final ensemble (3rd column). A comparison between the initial and final estimates of log PERMX and PORO indicates that the final estimates appear more similar to the reference fields, in consistence with the results in Fig 7.

### Results of scenario S2 (using seismic data only)

To examine the impact of data size on the performance of SHM, we consider two cases with different threshold values chosen through Eq (16). In the first case, we let *c* = 1, such that Eq (16) reduces to the universal rule in choosing the threshold value [66]. Under this choice, the number of leading wavelet coefficients is 178332, around 2.5% of the original AVA data size (7.04 × 10^6^). In the second case, we increase the value of *c* to 5, such that the number of leading wavelet coefficients further reduces to 3293, which is more than 4000 times reduction in comparison to the original data size. Let **d**^*ref*^ be the reference data (without noise), and **d**^*rec*^ be the data reconstructed through an inverse DWT using the leading wavelet coefficients of the noisy data **d**^*o*^ (while setting the rest of the wavelet coefficients of **d**^*o*^ to zero). Then to evaluate information loss in the sparse representation procedure, we adopt the following measure

$$L=\frac{\|d^{ref}-d^{rec}{\|}_{2}}{\|d^{ref}{\|}_{2}}\times 100\%.$$ (26)

In our experiments, the information loss is 38.7% when *c* = 1, whereas it creases to 91.4% at *c* = 5. The gap in information loss with different *c* values will be further illustrated later.

**Fig 10:**
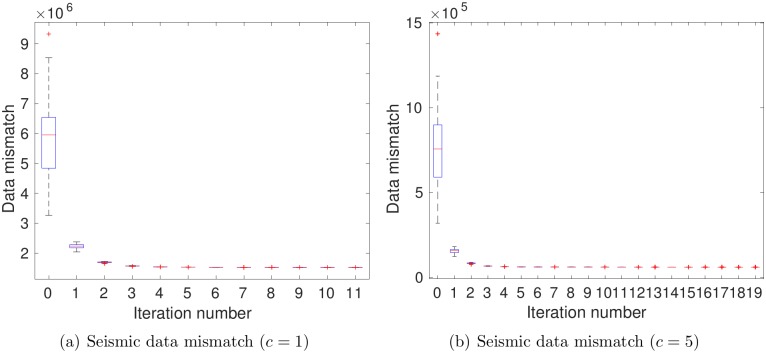
Boxplots of seismic data mismatch as functions of iteration step (scenario S2). Case (a) corresponds to the results with *c* = 1, for which choice the number of leading wavelet coefficients is 178332, roughly 2.5% of the original data size; Case (b) to the results with *c* = 5, for which choice the number of leading wavelet coefficients is 3293, more than 2000 times reduction in data size.

Fig 10 indicates the boxplots of seismic data mismatch as functions of iteration step. In either case, seismic data mismatch reduces fast at the first few iteration steps, and then changes slowly afterwards. The stopping criterion (C2), monitoring the relative change of average data mismatch, becomes effective in both cases, such that the iteration stops at the 11th step when *c* = 1, and at the 19th when *c* = 5. Accordingly, the ensembles at iteration step 11 and 19, respectively, are taken as the final estimates in these two cases. In addition, it appears that ensemble collapse takes place in both cases, although this phenomenon is somewhat mitigated in the case *c* = 5, in comparison to the case *c* = 1. The mitigation of ensemble collapse is even more evident when we further increase *c* to 8, and accordingly, reduce the data size to 534. By doing so, however, the history matching performance is deteriorated (results not included here), largely due to the fact that such a significant reduction of data size leads to substantial loss of information content in the seismic data, a point to be elaborated soon.

**Fig 11:**
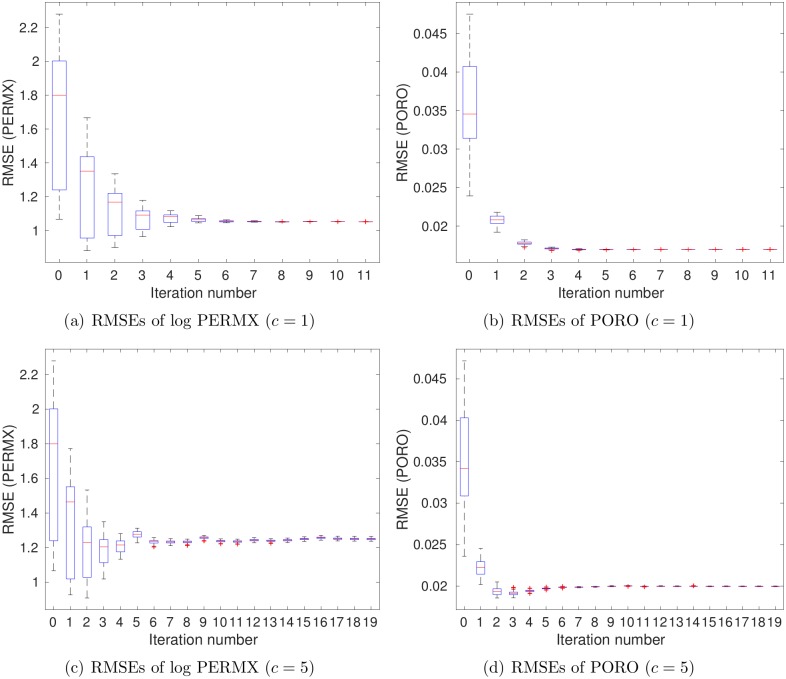
Boxplots of RMSEs of log PERMX (1st column) and PORO (2nd column) as functions of iteration step, with *c* being 1 (top) and 5 (bottom), respectively (scenario S2).

Fig 11 shows boxplots of RMSEs of log PERMX (1st column) and PORO (2nd column) as functions of iteration step. It is clear that the RMSEs of the final ensembles are lower than those of the initial ones, even at *c* = 5, the case in which data size is reduced more than 2000 times. On the other hand, when *c* = 1 (top row), the RMSEs of both log PERMX and PORO in the final ensemble are lower than those at *c* = 5 (bottom row). This indicates that, better history matching performance is achieved at *c* = 1, with more information content captured in the leading wavelet coefficients.

**Fig 12:**
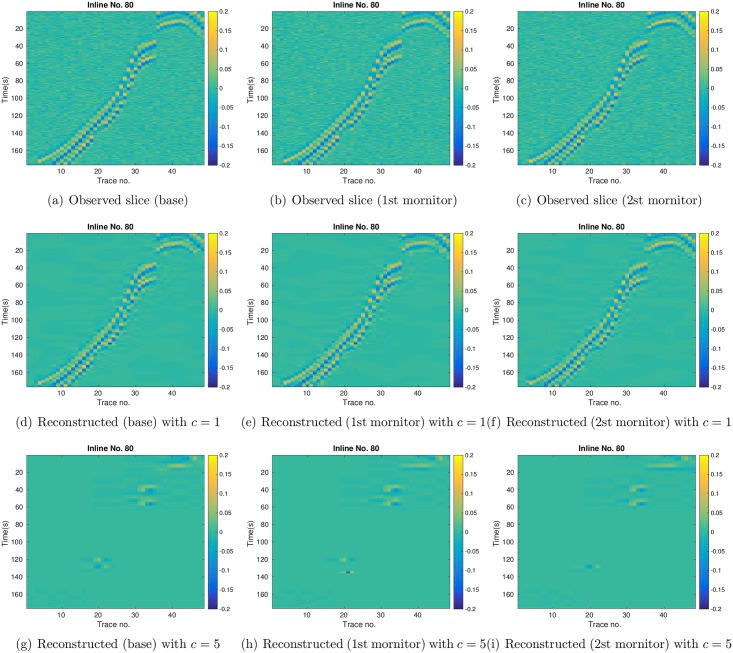
Top row: slices of the observed far-offset AVA attributes at *X* = 80, with respect to the base survey (1st column), the 1st monitor survey (2nd column) and the 2nd monitor survey (3rd column), respectively. Middle row: corresponding reconstructed slices at *X* = 80 using the leading wavelet coefficients at *c* = 1 (while all other wavelet coefficients are set to zero). Bottom row: corresponding reconstructed slices at *X* = 80 using the leading wavelet coefficients at *c* = 5 (while all other wavelet coefficients are set to zero).

As aforementioned, each 3D seismic dataset is in the dimension of 139 × 48 × 176. For illustration, the top row of Fig 12 indicates the slices of far-offset AVA attributes at *X* = 80, with respect to the base survey (1st column), the 1st monitor survey (2nd column) and the 2nd monitor survey (3rd column), respectively, whereas the middle and bottom rows show the corresponding slices reconstructed using the leading wavelet coefficients (while setting other coefficients to zero) at *c* = 1 and *c* = 5, respectively. Compared to figures in the top row, it is clear that the reconstructed ones at *c* = 1 capture the main features of the observed slices, while removing the noise component. Therefore in this case, although the universal rule (corresponding to *c* = 1) still leads to a relatively large data size, it achieves a good trade-off between data size reduction and feature preservation. In contrast, at *c* = 5, the seismic data size is significantly reduced. However, the reconstructed slices in the bottom row only retain a small portion of the strips in the observed slices of the top row, meaning that the data size is reduced at the cost of losing substantial information content of the seismic data. Nevertheless, even with such an information loss, using the leading wavelet coefficients at *c* = 5 still leads to significantly improved model estimation in comparison to the initial ensemble, and this will become more evident when both production and seismic data are used in scenario S3.

**Fig 13:**
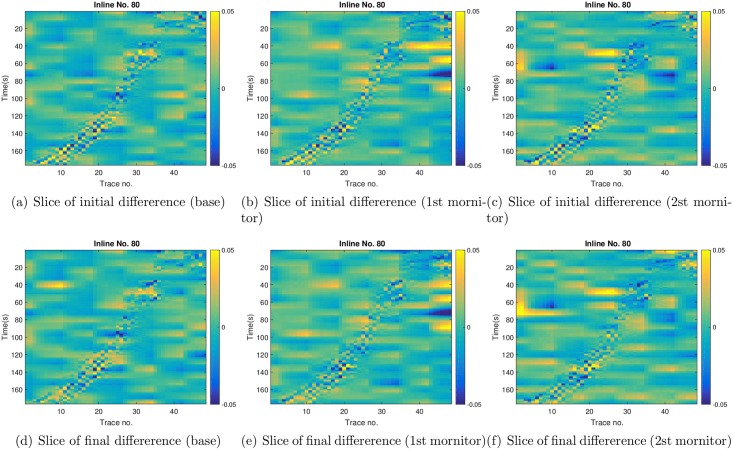
Top row: slices (at *X* = 80) of the differences between the reconstructed far-offset AVA attributes using the leading wavelet coefficients (*c* = 1) of the observed seismic data, and the reconstructed far-offset AVA attributes using the corresponding leading wavelet coefficients (*c* = 1) of the means of the simulated seismic data of the **initial** ensemble. From left to right, the three columns correspond to the differences at the base, the 1st monitor, and the 2nd monitor surveys, respectively. Bottom row: as in the top row, except that it is for the differences between the reconstructed far-offset AVA attributes of the observed seismic data, and the reconstructed far-offset AVA attributes of the mean simulated seismic data of the **final** ensemble.

For brevity, in what follows we only present the results with respect to the case *c* = 1. In the top row of Fig 13, we show the slices (at X = 80) of differences between two groups of reconstructed far-offset AVA attributes. One group corresponds to the reconstructed far-offset AVA attributes at three survey times, using the leading wavelet coefficients (*c* = 1) of the observed far-offset AVA attributes. The other group contains the reconstructed far-offset AVA attributes at three survey times, using the corresponding leading wavelet coefficients (*c* = 1) of the mean simulated seismic data of the initial ensemble. Therefore the slices of differences in the top row can be considered as a reflection of the initial seismic data mismatch in Fig 10A. Here, we use the slices of differences for ease of visualization, as the reconstructed slices of the observed and the mean simulated AVA attributes look very similar. Similarly, in the bottom row, we show the slices of differences between the reconstructed far-offset AVA attributes of the observed seismic data, and the reconstructed far-offset AVA attributes of the mean simulated seismic data of the final ensemble. In this case, the slices of differences can be considered as a reflection of the final seismic data mismatch in Fig 10A. Comparing the top and bottom rows at a given survey time, one can observe certain distinctions, which, however, are not very significant in general. This is in line with the results in Fig 10A, where the initial and final seismic data mismatch remain in the same order, in contrast to the substantial reduction of production data mismatch in scenario S1 (Fig 6).

**Fig 14:**
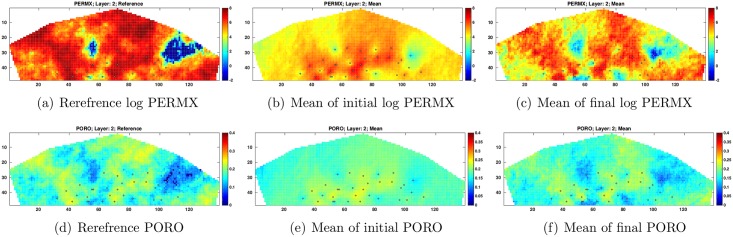
As in Fig 9, but for scenario S2 with *c* = 1.

Similar to Fig 9, Fig 14 depicts the reference log PERMX and PORO at layer 2 (1st column), the mean of initial log PERMX and PORO at layer 2 (2nd column), and the mean of final log PERMX and PORO at layer 2 (3rd column). Compared to the initial mean estimates, the final mean log PERMX and PORO show clear improvements, in terms of the similarities to the reference fields. In addition, an inspection on the 3rd columns of Figs 9 and 14 reveals that the final mean estimates in S2 capture the geological structures of the reference fields better, especially in areas where there is neither injection nor production well (well locations are represented by black dots in Figs 9 and 14).

### Results of scenario S3 (using both production and seismic data)

**Fig 15:**
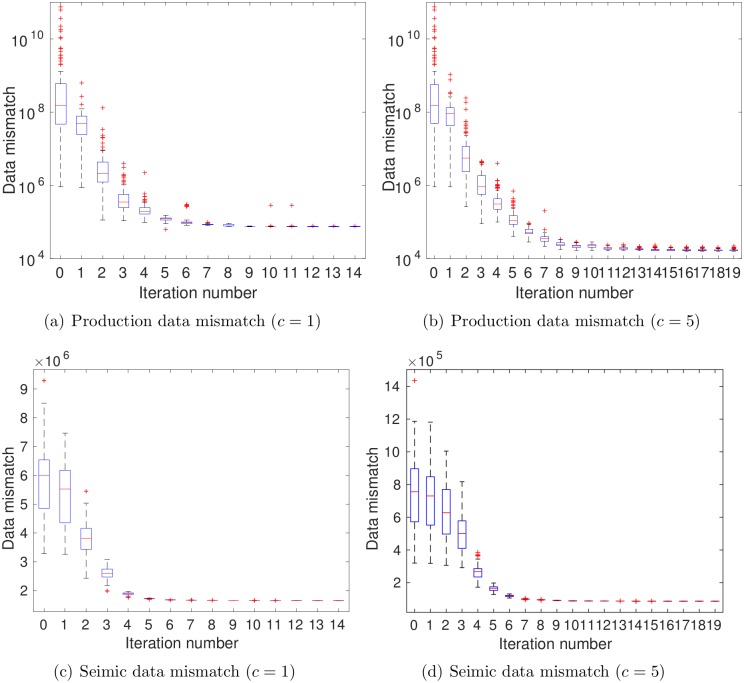
Boxplots of production (top) and seismic (bottom) data mismatch as functions of iteration step (scenario S3).

In scenario S3, production and seismic (in terms of leading wavelet coefficients) data are assimilated simultaneously. Fig 15 reports the boxplots of production (top) and seismic (bottom) data mismatch as functions of iteration step. Because of the simultaneous assimilation of production and seismic data, the way to use the seismic data (in terms of the value of *c* in Eq (16)) will affect the history matching results. This becomes evident if one compares the first and second columns of Fig 15. Indeed, when *c* = 1, because the relatively large data size, it is clear that ensemble collapse takes place in Fig 15A and 15C. Also, the iteration stops at step 14, due to the stopping criterion (C2). By increasing *c* to 5, the size of seismic data is reduced from 178332 to 3293, and ensemble collapse seems mitigated to some extent, especially for production data, while the final iteration step is 19, due to the stopping criterion (C2). On the other hand, by comparing Figs 6, 10 and 15, it is clear that, in S3, the presence of both production and seismic data makes the reduction of data mismatch different from the case of using either production or seismic data only. For instance, in the presence of seismic data, the production data mismatch (see Fig 15A and 15B) tend to be higher than that in Fig 6. On the other hand, with the influence of production data, the occurrence of ensemble collapse seems to be delayed in Fig 15C and 15D, in comparison to those in Fig 10.

**Fig 16:**
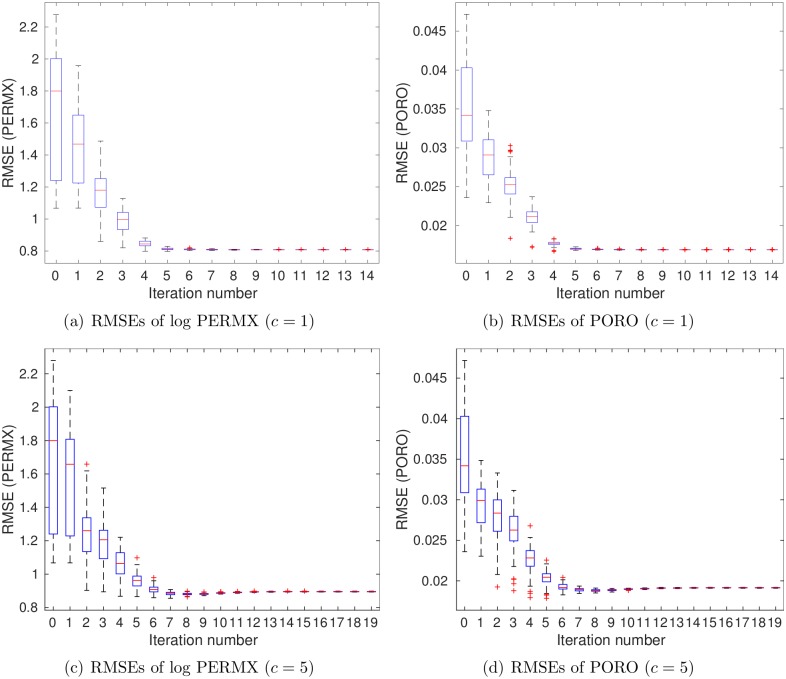
Boxplots of RMSEs of log PERMX (1st column) and PORO (2nd column) as functions of iteration step, with *c* being 1 (top) and 5 (bottom), respectively (scenario S3).

Fig 16 shows boxplots of RMSEs of log PERMX (1st column) and PORO (2nd column) as functions of iteration step. Again, the RMSEs of the final ensembles are lower than those of the initial ones, for either *c* = 1 or *c* = 5. On the other hand, a comparison of Figs 7, 11 and 16 indicates that the RMSEs of log PERMX and PORO (and similarly, the RMSEs of log PERMY and log PERMZ) are the lowest when using both production and seismic data in history matching. Using *c* = 5 in scenario S3 (Fig 16C and 16D) leads to higher RMSEs than using *c* = 1. Nevertheless, they are still better than the RMSEs in scenario S1, and close to (for PORO) or better than (for log PERMX) those in scenario S2 with *c* = 1 (see Fig 11A and 11B). This suggests that, in this particular case, reasonably good history matching performance can still be achieved, even though the data size is reduced more than 2000 times (at *c* = 5) through the wavelet-based sparse representation procedure.

**Fig 17:**
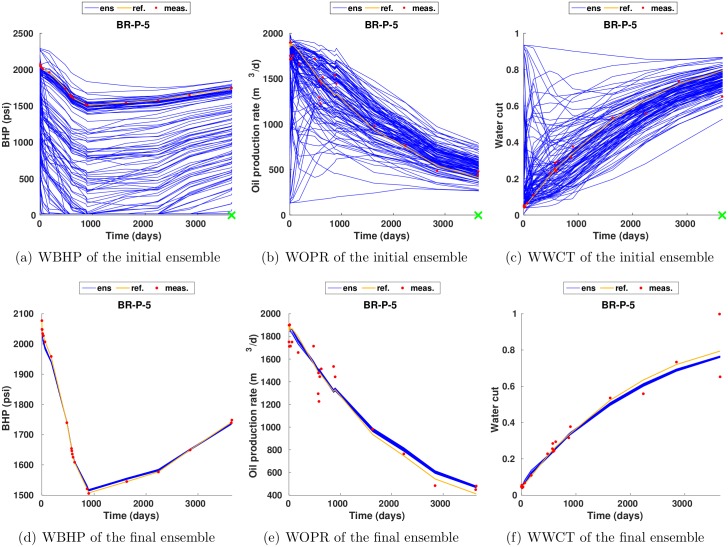
As in Fig 8, but for the production data profiles in scenario S3 with *c* = 1.

Again, for brevity, in what follows we only report the results with respect to the case *c* = 1. Fig 17 shows the production data profiles at the producer BR-P-5 in scenario S3 with *c* = 1. Clearly, compared to the initial ensemble, the final one matches the observed production data (red dots) better. Nevertheless, a comparison of the bottom rows of Figs 8 and 17 indicates that, the ensemble spreads of simulated production data (blue curves) tend to be under-estimated, such that the reference production data (yellow curves) are outside the profiles of simulated production data at certain time instances.

**Fig 18:**
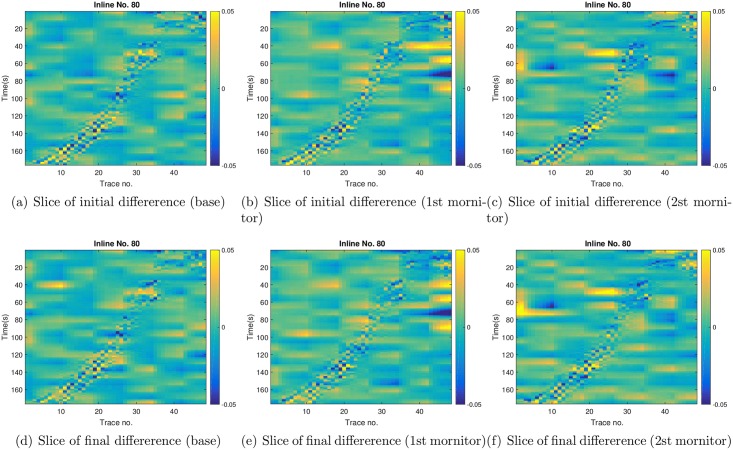
As in Fig 13, but for the slices (at *X* = 80) of differences in scenario S3 with *c* = 1.

Similar to Fig 13, in Fig 18 we show the slices (at X = 80) of differences between the reconstructed far-offset AVA attributes of observed and mean simulated seismic data, at three survey times. Again, compared to the slices with respect to the initial ensemble (top), there are some visible distinctions in the slices with respect to the final ensemble (bottom). However, if one compares the bottom rows of Figs 13 and 18, it seems that these slices look very similar to each other. This is consistent with the results in Figs 10A and 15C, where the final seismic data mismatch of S2 and S3 remains close.

**Fig 19:**
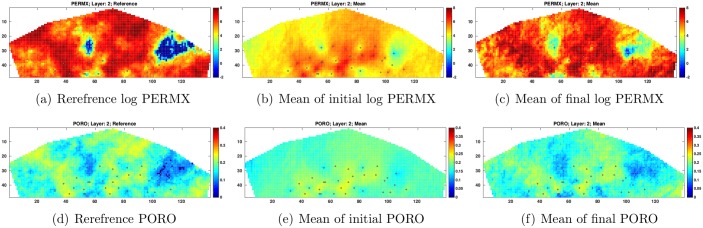
As in Fig 9, but for scenario S3 with *c* = 1.

Finally, Fig 19 compares the reference, initial and final mean log PERMX and PORO fields at layer 2. Again, the final mean estimates improve over the initial mean fields, in terms of the closeness to the references. In addition, a comparison of the final estimated fields (the 3rd columns) of Figs 9, 14 and 19 shows that the final mean estimates in S3 best capture the geological structures of the reference fields (the same observation is also obtained at *c* = 5). This indicates the benefits of using both production and seismic data in history matching.

## Discussion and conclusion

In this work, we extend a 2D wavelet-based sparse representation procedure used in [8] to handle 3D seismic datasets, and integrate it into an ensemble-based seismic history matching framework. To demonstrate the efficiency of the integrated workflow, we apply it to a 3D benchmark case, the Brugge field case. The seismic data used in this study are near- and far-offset amplitude versus angle (AVA) attributes, with the data size being more than 7 million.

Through numerical experiments, we show that the 3D sparse representation procedure is very efficient for data-size reduction, and works well for noise STD estimation in the wavelet domain. Via sparse representation, the size of seismic data (in the form of leading wavelet coefficients) can be conveniently controlled through a threshold value. A relatively large threshold value means more reduction in data size, which is desirable for the history matching algorithm, but at the cost of extra information loss. In contrast, a relatively small threshold value results in a larger number of leading wavelet coefficients, hence better preserves the information content of observed data. In this case, however, the history matching algorithm may become more vulnerable to certain practical issues like ensemble collapse. As a result, the best practice would need to achieve a trade-off between reduction of data size and preservation of data information. Another observation from the experiment results is that, in this particular case, a combined use of production and seismic data in history matching leads to better estimation results than the cases of using either production or seismic data only.

Ensemble collapse is clearly visible when seismic data is used in history matching. This phenomenon can be mitigated to some extent by increasing the threshold value (hence reducing the seismic data size), but it cannot be completely avoided. A possible remedy to this problem is to also introduce localization (see, for example, [69, 70]) to the iterative ensemble smoother. In the presence of the sparse presentation procedure, however, seismic data are transformed into the wavelet domain, and the concept of “physical distance” are not valid any more. As a result, localization will need to be adapted to this change. We will investigate this issue in our future study.

In the present study, the AVA datasets are 3D attributes, and there are no missing data points. In this case, the datasets exhibit regular spatial coverages, and the conventional wavelets can be directly used. When extending the sparse representation procedure to other types of datasets, e.g., acoustic impedance distributed over active reservoir gridblocks and remotely-sensed ocean or atmosphere satellite images, the datasets of interest may have irregular spatial coverages. In this case, due to the irregularities of data distribution fields, the conventional wavelets may not be applicable. To address this issue, there are two possible strategies. One is to add some artificial data (e.g., mean field values) in such a way that, with the extra data points, the expanded datasets have regular spatial coverages. The other (more sophisticated) strategy is to modify the lifting scheme in the wavelets, such that the corresponding DWT is applicable to datasets with irregular spatial distributions [71]. We have tested the first simple idea (adding artificial data points), and observed that it works in some preliminary studies (results not reported here). Implementing the second idea (modifying the wavelet lifting scheme) is more complicated in general, and has not been fully investigated by us yet.

In practice, the observation errors of certain seismic data, such as (inverted) acoustic impedance, may exhibit spatial correlations and thus should be treated as colored noise. In this case, it may not be appropriate to only use the wavelet coefficients in the sub-band resulted from the high-pass filters to estimate the noise level. Instead, it is advocated to estimate the noise level for each wavelet sub-band [40, 41]. Based on this notion, an enhancement of the wavelet-based sparse representation procedure used in the current study will be implemented to handle seismic data with colored noise, and the relevant results will be reported elsewhere.
